# The Association of Human Milk Appetite-Regulating Hormones with Infant Growth and Eating Behaviors to Age Six Months

**DOI:** 10.3390/nu18081203

**Published:** 2026-04-10

**Authors:** Adrienne Bruder, Lindsay Ellsworth, Julie Sturza, Brigid Gregg, Alison L. Miller, Julie C. Lumeng

**Affiliations:** 1 Department of Pediatrics, University of Michigan, Ann Arbor, MI 48109, USA; lindsay_ellsworth@ihacares.com (L.E.); jlumeng@med.umich.edu (J.C.L.); 2 Division of Neonatology, Department of Pediatrics, University of Minnesota, Minneapolis, MN 55454, USA; 3 Trinity Health Ann Arbor Hospital, Ypsilanti, MI 48197, USA; 4 Division of Pediatric Endocrinology, Department of Pediatrics, University of Michigan, Ann Arbor, MI 48109, USA; 5 Department of Health Behavior & Health Education, School of Public Health, University of Michigan, Ann Arbor, MI 48109, USA; alimill@umich.edu

**Keywords:** human milk, whole human milk, term breastfed infants, appetite-regulating hormones, leptin, adiponectin, breastfeeding intensity, anthropometry

## Abstract

**Background/Objectives**: Appetite-regulating hormones are bioactive components of human milk. We tested the associations of leptin and adiponectin with infant growth and eating behaviors to age 6 months. **Methods**: In a cohort of 70 healthy, full-term infants and their mothers, human milk adiponectin and leptin were assayed at age 2 months (m). At infant ages 2, 4, and 6 m, infant anthropometry was obtained, mothers reported feeding frequency, duration, and breastfeeding intensity and completed the Baby Eating Behavior Questionnaire (Enjoyment of Food, Food Responsiveness, and General Appetite), and infant sucking vigor using an artificial nipple (burst duration and sucking frequency) was measured. Mothers reported demographics, gestational diabetes and pre-pregnancy body mass index (BMI), gestational age, and infant birthweight. Multivariate models evaluated predictors of leptin and adiponectin, and associations of leptin and adiponectin with infant growth and eating behaviors. **Results**: Human milk leptin was predicted by maternal BMI (β = 0.02) and breastfeeding intensity (β = −0.32). Regarding infant growth, infant weight-for-age and weight-for-length z-scores at 6 m were predicted by leptin (β = 0.91 and β = 1.22, respectively) and adiponectin (β = 0.01 and β = 0.01, respectively). Regarding infant eating behaviors, feeding duration at 2 m and feeding frequency at 4 m were predicted by adiponectin (β = 0.03 and β = −0.02, respectively). **Conclusions**: Human milk leptin and adiponectin may contribute to weight gain in early infancy, but the effect does not appear to be mediated substantially by infant eating behaviors. Further investigation into the metabolic programming of early infant weight gain is warranted.

## 1. Introduction

Rate of weight gain in early infancy has been associated with childhood and adulthood obesity [1]. The mechanisms of early infant weight gain, however, remain poorly understood. Appetite-regulating hormones in human milk (HM), including leptin and adiponectin, have been theorized to contribute to infant growth among breastfed infants on the premise that leptin is anorexigenic and adiponectin is orexigenic [2,3,4,5,6,7]. Findings of prior studies testing associations of HM hormones with infant growth have been mixed [8]. Interpretation of these prior studies has been challenging due to several limitations [5,8]. First, studies have often not accounted for the proportion of the infant’s diet that is HM versus formula. This is relevant both due to dose effects as well as due to reported associations between HM hormones and the amount of milk produced [9,10,11]. Second, prior studies have not controlled for potentially important covariates, including maternal age and body mass index (BMI), and infant age, sex, and birthweight [2,8]. Third, infant anthropometry and HM samples have not always been collected using standardized approaches [5,8]. Fourth, approaches to assaying HM have often been described in limited detail or used suboptimal approaches [3,8,12]. This study therefore sought to examine associations of HM hormones with infant growth while addressing these methodological limitations.

Given that leptin is anorexigenic and adiponectin is orexigenic, HM leptin would be hypothesized to positively associate with intake-inhibiting and negatively associate with intake-promoting eating behaviors, while adiponectin would be hypothesized to positively associate with intake-promoting and negatively associate with intake-inhibiting eating behaviors [6,7]. Such eating behaviors are expected to be precursors to infant growth. Support for these hypotheses comes from animal models showing the expected associations between appetite-regulating hormones in milk and 24 h intake, preference for energy-dense food, and feeding size [13,14,15]. Results in human studies have been more mixed; in infancy, associations of HM leptin have been null or positive for measures of eating behaviors [16,17,18,19]. Likewise, associations of HM adiponectin have been null or positive for intake-promoting eating behaviors [16,19,20]. Sucking vigor is another intake-promoting eating behavior that can be reliably measured in infancy, but we have been unable to identify any studies testing associations with HM hormones [21,22]. This study therefore sought to examine associations of HM hormones with previously unexamined eating behaviors that are known precursors to infant growth.

This study’s objective was therefore to examine associations of HM leptin and adiponectin with infant growth and eating behaviors to age six months while addressing several methodological limitations of prior work. Based on the prior literature, we hypothesized that leptin would negatively associate and adiponectin would positively associate with infant anthropometry and growth-promoting eating behaviors.

## 2. Materials and Methods

### 2.1. Study Design

The overall study sought to examine the development of infant eating behavior longitudinally within participants at ages 1, 2, 4, 6, 9, and 12 months based on data collected from questionnaires, eating behavior experiments, and anthropometry via home visits with trained research staff. Participants were enrolled between October 2015 and February 2019.

### 2.2. Participants and Recruitment

A convenience sample of mother–infant dyads was recruited by advertising in communities within a 1.5 h driving distance of Ann Arbor, MI, USA, via flyers, social media advertisements, and targeted phone, email, and mail outreach to pregnant women and mothers of newborns. To facilitate recruitment, infants could enroll at ages 1, 2, or 4 months.

When mothers expressed interest in the study, research staff connected with the mother by telephone and performed screening using a scripted protocol. The study was described as completely voluntary, the study purpose was explained as seeking to understand infant eating behavior and mother–infant interactions in the first year, and planned study activities were explained in detail. Mothers who were interested were screened for eligibility. Inclusion criteria for the overall study were: gestational age 37.0 to 42.0 weeks, weight appropriate for gestational age, no significant perinatal or neonatal complications, biological mother was legal and custodial guardian, and infant had consumed two ounces or more in one feeding from an artificial nipple at least once per week. Human milk sample collection at age 2 months had the additional inclusion criterion that mothers were breastfeeding. Exclusion criteria for the overall study were: mother not fluent in English, mother <18 years, infant medical problems or diagnosis affecting eating, growth, or development, or child protective services involvement.

### 2.3. Data Collection Method

Mother–infant dyads who were eligible scheduled their first data collection home visit. Infant anthropometry was completed at the first study visit and was required before any additional study activities could be offered. All participants were invited to participate in all study activities at every age, though only a subset of participants were invited to have sucking measured or human milk samples collected, given that these study activities were added after some participants had already enrolled.

### 2.4. Anthropometry

At ages 2, 4, and 6 months, infant weight and length were measured by research staff certified in appropriate measurement techniques by a certified trainer [23]. Infants were weighed without clothing or a diaper on a BD-585 Digital Infant Scale (Tanita, Tokyo, Japan) in duplicate, and were weighed a third time if weights differed by more than 0.1 kg; subsequently, the two most similar weights were averaged. Using a pediatric stadiometer (M-PED LB 35-107-X; Ellard Instrumentation Ltd., Monroe, WA, USA), recumbent length was measured in duplicate to the nearest 0.1 cm, and a third time if lengths differed by more than 0.2 cm; subsequently, the two most similar lengths were averaged. Using the World Health Organization growth standards, we generated z-scores for weight-for-age (WAZ), length-for-age (LAZ), and weight-for-length (WLZ) [24].

### 2.5. Eating Behaviors

Mothers reported infant eating behaviors at ages 2, 4, and 6 months. Mothers completed the Infant Feeding Practices Study II questionnaire (IFPS II) [25]. Mothers reported feeding frequency and average breastfeeding duration (Supplemental Materials). Breastfeeding intensity was calculated from these data as the number of human milk feeds divided by the total number of liquid feeds (both human milk feeds and formula feeds), as defined by others previously [26].

Mothers responded to the 18-item Baby Eating Behavior Questionnaire (BEBQ), which is designed to assess formula or milk feeding behaviors in infancy [27]. Items are rated on a 5-point Likert scale (never = 1; rarely = 2; sometimes = 3; often = 4; always = 5) and mean scores are calculated after reverse scoring as appropriate to generate four subscales. Across cohorts internationally, the internal consistency of the Satiety Responsiveness and Slowness in Eating subscales has varied widely, frequently with a Cronbach’s α < 0.60. Our cohort followed a similar pattern, and we therefore focused on Enjoyment of Food (4 items, α = 0.61, 0.70, and 0.67 at 2, 4, and 6 months, respectively) and Food Responsiveness (6 items, α = 0.77, 0.78, and 0.77 at 2, 4, and 6 months, respectively), as well as the single item General Appetite scale. Parental ratings for these three subscales show significant within-subject correlation across age-points in early infancy, positively correlate with adiposity across multiple cohorts internationally, and show significant heritability [28,29,30].

At infant ages 2, 4, and 6 months, mothers were asked to feed their infant a typical bottle feeding of formula or human milk, in her usual manner, when the infant was hungry. Details of this protocol are reported elsewhere [31]. Briefly, the bottle was equipped with an NFANT^®®^ Feeding Solution (NFANT Labs, Atlanta, GA, USA), which captures sucking with a cantilever mechanism and transforms the resulting pressure wave to sucking metrics, which are averaged across the feeding [32,33,34]. In full-term, healthy infants, nutritive sucking is composed of a burst–pause pattern in which sucks occur at a rate of about one suck per second in series of varying durations (i.e., a sucking burst), followed by a pause of at least two seconds before the next burst. We examined burst duration and sucking rate, as these are commonly viewed as indices of sucking vigor and are positively independently associated with infant intake and weight gain [21,22,35]. Burst duration was the time in seconds from the start of first suck to the end of last suck in a series of at least three sucks, with inter-suck intervals of less than two seconds. Sucking rate was the number of sucks per second within a burst. Mother-reported time elapsed since the last feeding, amount consumed at the last feeding, and the type of milk/formula used were not related to either burst duration or sucking rate and were therefore not considered further in analyses. In May 2017, a change in available equipment necessitated a change in the device used to measure sucking and therefore sucking data was only included after this change.

### 2.6. Human Milk Collection and Hormone Assays (Primary Outcome and Predictor)

HM samples were collected at two months postpartum. Mothers were provided written instructions. Mothers were asked to use their personal breast pump to express a single entire breast. They were asked to continue to pump milk until only drops were expressed for a full minute. Mothers were asked to not have fed the infant from the breast selected for pumping in the last two hours, and to obtain the sample between 8 and 10 am. The samples were obtained, on average, at 8:55 (SD 0:55, median 8:50, interquartile range 8:15 to 9:32, range 6:00–12:30). Time of sample collection was not associated with either leptin (r = −0.05, *p* = 0.70) or adiponectin (r = 0.08, *p* = 0.53) and was not considered further as a covariate in analyses. Mothers were instructed to gently shake the bottle to mix the milk well and then use a study-provided transfer pipet to aliquot milk into five 5 mL glass vials. Milk samples were stored in a plastic bag in the mother’s freezer until a research staff member who had completed institutionally mandated biosafety training retrieved them and transported them on ice to the laboratory. Samples were then stored at −80 °C until analysis in a locked research laboratory. Biosafety level 2 protocols were followed for all aspects of transport, handling, manipulating, and disposing of human milk.

Human milk samples were only assayed for participants who had complete data available for the planned analyses. Specifically, dyads were required to have complete data for anthropometry at 2, 4, and 6 months, as well as BEBQ subscales and sucking measures at 2 months. All analyses were performed on whole milk, as previously described, validated, and recommended to most accurately assess leptin content [12,36]. A 5 mL vial was thawed and 250 μL aliquots were refrozen. A 250 μL aliquot of whole milk was thawed on ice and then sonicated using a Qiagen TissueLyser II Bead Homogenizer (Qiagen, Hilden, Germany) (three cycles of 5 s, frequency 30 Hz). The leptin protocol was previously validated for use on whole HM using a Human Leptin Enzyme-Linked Immunosorbent Assay (ELISA) (R&D Systems, Minneapolis, MN, USA) with a dilution factor of 10 [12]. Adiponectin was measured in whole HM using a Biovendor Human Adiponectin Sandwich ELISA kit and following the protocol included (Life Technologies, Asheville, NC, USA), as previously described [37]. ELISA measurements were performed in duplicate, with the average value used in analysis.

### 2.7. Covariates

Mothers self-reported birth date, from which maternal age was calculated. Maternal metabolic status was conceptualized as having had a diagnosis of gestational diabetes and pre-pregnancy BMI. Mothers reported whether they had received a diagnosis of gestational diabetes during the pregnancy with the index child (yes = 1 vs. no = 0). Mothers self-reported their pre-pregnancy weight. Height was measured using a portable stadiometer (Seca 213/217, Seca, Hamburg, Germany) to calculate pre-pregnancy body mass index (BMI). As a marker of socioeconomic status, mothers reported their level of education, which was subsequently categorized for analysis into an ordinal 3-level variable: ≥graduate degree (=3), 4-year undergraduate degree (=2), or <4-year undergraduate degree (=1).

Mothers reported their due date and infant’s birth date, from which infant age and gestational age were calculated, as well as the infant’s birthweight and sex (female = 1, male = 0). Mothers reported their own and the infant’s race/ethnicity for descriptive purposes. Response options were those used by the United States National Institutes of Health (American Indian or Alaska Native, Asian, Black or African American, Native Hawaiian or Pacific Islander, White, Multiracial, and Other, as well as Hispanic or Latino).

### 2.8. Statistical Analysis

Univariate statistics were used to describe the sample. Pearson’s correlation was used to assess the associations of cohort characteristics which were being considered for inclusion in multivariate models with each primary outcome (milk hormone concentrations and anthropometry) and each secondary outcome (eating behaviors). Simple linear regression was used to first test the unadjusted associations of leptin and adiponectin independently in separate models with each anthropometry or eating behavior outcome, adjusting for breastfeeding intensity to account for the ‘dose’ of leptin and adiponectin. Multivariate linear regression was used to test the associations of leptin and adiponectin in separate models, again adjusting for breastfeeding intensity to account for the ‘dose’, and also adjusting for the other cohort characteristics previously identified as being associated with each anthropometric or eating behavior outcome. Separate models were run for each age point. Results are reported as beta coefficients (β) with standard errors with corresponding *p*-values. Analyses were performed using SAS 9.4 and GraphPad Prism 10.4.1.

### 2.9. Ethics

At the first home visit, before any study activities occurred, the informed consent was reviewed in detail, including that the study was voluntary, minimal risk, and provided no direct benefit to participants. Mothers provided written informed consent for themselves and their infants. The study was approved by the University of Michigan institutional review board (HUM00103575, approved 28 August 2015).

## 3. Results

### 3.1. Cohort Characteristics

**Figure 1:**
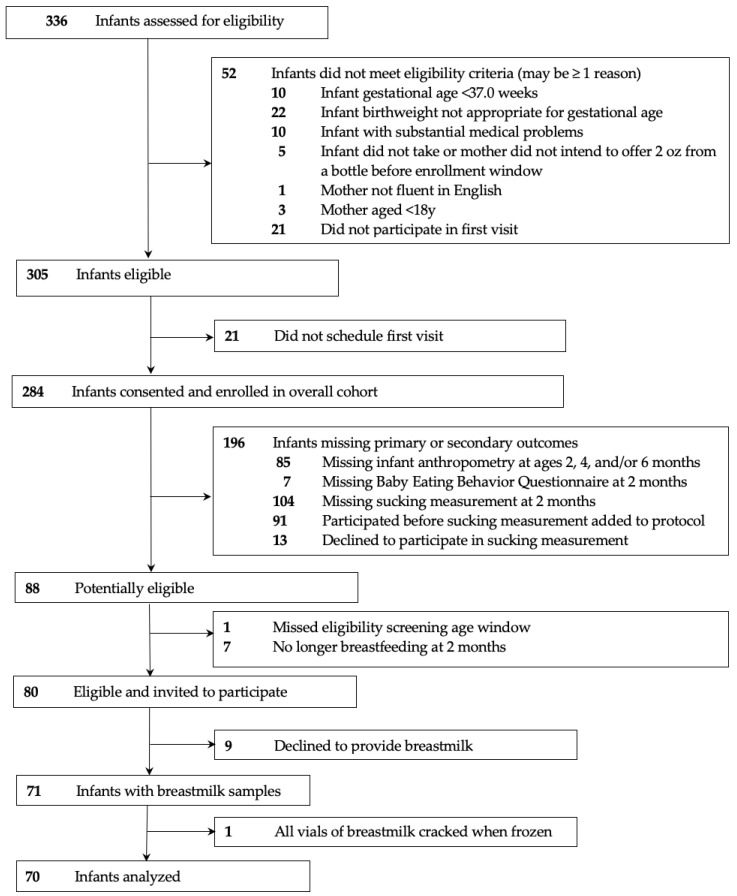
Participant flow diagram.

The participant flow diagram detailing the identification of the analytic sample is shown in Figure 1. A total of 284 mother–infant dyads consented and enrolled in the overall study. Of these, 80 had the complete anthropometry and eating behavior data required for inclusion in the analysis and were still breastfeeding at age two months. Of these, 71 (89%) provided HM samples and 70 infants had HM that was successfully assayed. Differences between the analytic and excluded samples are shown in Supplementary Table S1.

Characteristics of the sample of 70 infants and their mothers are shown in Table 1. The cohort of mothers had a mean age of 31.5 years (SD 5.0) and mean pre-pregnancy BMI of 27.2 (SD 5.9); 11% had gestational diabetes; 74% were non-Hispanic white; 9% non-Hispanic black; 1% Hispanic of any race; and 17% non-Hispanic and another race or multiracial.

The cohort of infants had a mean gestational age of 39.4 weeks (SD 1.05) and mean birthweight of 3.43 kg (SD 0.36); 56% were female; and 73% had a breastfeeding intensity of 1.0, indicating exclusive HM feeding. Mean infant age at the 2-month HM collection was 2.3 (SD 0.4), range 1.6 to 3.4 months. Mean infant ages at the 2-, 4-, and 6-month anthropometry collections were 2.2 (SD 0.4), range 1.4 to 3.4; 4.3 (SD 0.5), range 3.6 to 5.4; and 6.4 (SD 0.5), range 5.7 to 8.0.

**Figure 2:**
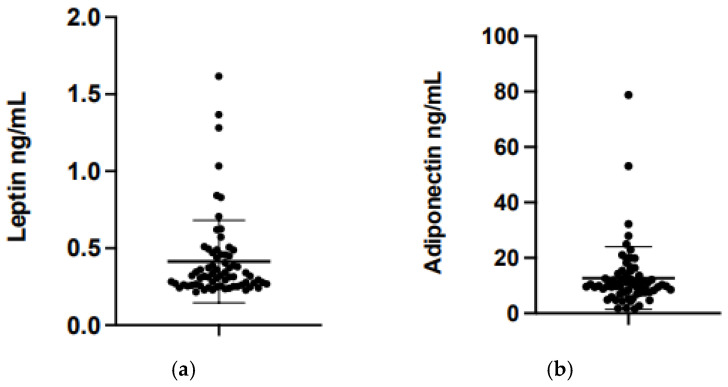
(**a**) Mean milk leptin concentration was 0.41 ng/dL (SD 0.27), while (**b**) adiponectin was 14.31 ng/dL (SD 17.33).

HM hormone concentrations are shown in Table 1 and Figure 2. Mean leptin was 0.41 ng/mL (SD 0.27) and mean adiponectin was 14.31 ng/mL (SD 17.33). Mean WAZ at 2, 4 and 6 months was −0.21, −0.22, and −0.11, respectively, and WLZ at 2, 4, and 6 months was 0.06, 0.07, and 0.35, respectively. Mean change in WAZ and WLZ from 2 to 6 months was 0.03 and 0.07, respectively. Mean LAZ at 2, 4, and 6 months and change in LAZ from 2 to 6 months are shown in Supplementary Table S2. With regard to the eating behavior outcomes at age 2 months, mean feeding frequency was 7.6 (i.e., 7–8 times per day), mean feeding duration was 2.7 (i.e., 10 to 30 min), mean Enjoyment of Food was 4.5, Food Responsiveness 2.1, General Appetite 3.9, Suck Frequency 1.53 Hz, and Burst Duration 37.7 s. Means for eating behavior outcomes at 4 and 6 months are shown in Supplementary Table S3.

### 3.2. Correlations of Cohort Characteristics with Human Milk Hormones, Anthropometry, and Eating Behaviors

Correlations of cohort characteristics with HM hormones, anthropometry, and eating behaviors are shown in Table 1. Positive correlations included pre-pregnancy BMI and leptin (*p* = 0.001), birthweight and WAZ at 2 months (*p* < 0.0001), birthweight and WAZ at 4 months (*p* < 0.0001), birthweight and WAZ at 6 months (*p* < 0.0001), pre-pregnancy BMI and WLZ at 6 months (*p* = 0.04), birthweight and WLZ at 6 months (*p* = 0.02), pre-pregnancy BMI and ∆ WAZ from 2 to 6 months (*p* = 0.004), and pre-pregnancy BMI and ∆ WLZ from 2 to 6 months (*p* = 0.002). Negative correlations included breastfeeding intensity and leptin (*p* = 0.04), maternal age and adiponectin (*p* = 0.04), breastfeeding intensity and adiponectin (*p* = 0.04), maternal education and WLZ at 2 months (*p* = 0.02), female infant sex and WLZ at 6 months (*p* = 0.02), female infant sex and ∆ WLZ from 2 to 6 months (*p* = 0.008), breastfeeding intensity and feeding duration (*p* = 0.02), maternal age and Enjoyment of Food (*p* = 0.04), maternal education and Enjoyment of Food (*p* = 0.002), female infant sex and Enjoyment of Food (*p* = 0.007), maternal education and Food Responsiveness (*p* = 0.002), maternal education and General Appetite (*p* = 0.002), and female infant sex and General Appetite (*p* = 0.001). All other correlations tested were not significant.

### 3.3. Associations of Human Milk Hormone Concentrations with Infant Anthropometry

Models testing associations of leptin and adiponectin with anthropometry outcomes are shown in Table 2. In models adjusted only for breastfeeding intensity, leptin was positively associated with WLZ at 4 months (*p* = 0.048) and 6 months (*p* = 0.01) and ∆ WAZ from 2 to 6 months (*p* = 0.003) and adiponectin was positively associated with WLZ at 6 months (*p* = 0.02). After adjusting for the additional cohort characteristics previously identified as being associated with each anthropometric outcome in correlation analyses in Table 1, leptin was positively associated with WAZ at 6 months (*p* = 0.03), WLZ at 6 months (*p* = 0.01), and ∆ WAZ from 2 to 6 months (*p* = 0.04) and adiponectin was positively associated with WAZ at 6 months (*p* = 0.048) and WLZ at 6 months (*p* = 0.04). No significant associations of leptin or adiponectin with any LAZ outcome were identified (Supplementary Table S2).

### 3.4. Associations of Human Milk Hormone Concentrations with Infant Eating Behaviors

Models testing associations of leptin and adiponectin with eating behavior outcomes at age 2 months are shown in Table 3. In the models adjusted only for breastfeeding intensity, leptin was not associated with any eating behavior outcome while adiponectin was positively associated with feeding duration (*p* = 0.03). After adjusting for the additional cohort characteristics previously identified as being associated with each eating behavior outcome in the correlation analyses in Table 1, leptin continued to not be associated with any eating behavior outcome and adiponectin remained positively associated with feeding duration at 2 months (*p* = 0.03).

Models testing associations of leptin and adiponectin with eating behavior outcomes at ages 4 and 6 months are shown in Supplementary Table S3. In the models adjusted only for breastfeeding intensity, leptin was not associated with any eating behavior outcome at either 4 or 6 months while adiponectin was negatively associated with feeding frequency at 4 months. Results did not substantially change after adjusting for the additional cohort characteristics previously identified as being associated with each eating behavior outcome.

## 4. Discussion

This study had two main findings. First, HM leptin and adiponectin at 2 months were positively associated with infant adiposity at 6 months but not at earlier ages and not with linear growth. While these null associations are consistent with the majority of prior literature, the emergence of positive associations with adiposity at older ages differs from the null to weakly negative associations reported in most prior studies [8]. Our study addressed several of the methodological limitations making prior studies difficult to interpret, however, and positive associations have been reported in other studies that have similarly addressed these methodological limitations [19,38,39].

The second main finding of this study was that HM leptin and adiponectin did not associate with any of the eating behaviors measured at any age, with the exception of HM adiponectin positively associating with feeding duration at 2 months and negatively associating with feeding frequency at 4 months. This is consistent with most prior literature, [16,17,18,20,40]. The fact that we detected few associations with eating behaviors may be due to limitations of measurements, including that they were either parent-reported or reflected only a single feeding. Our study may also not have had sufficient power to detect a modest effect size. Overall, however, the findings suggest that the mechanisms through which HM adiponectin and leptin impact infant weight gain are through pathways other than eating behavior. Lactational programming would posit that if the mechanisms were unrelated to changes in ingested milk volume, the milk hormones may alter caloric absorption or metabolism. This could occur locally via intestinal inflammation or systemically via fatty acid or glucose and insulin signaling. Both leptin and adiponectin have demonstrated roles inhibiting insulin secretion while also enhancing insulin action, which warrants additional investigation and may help explain the observed findings related to promoting infant growth [41].

Associations between covariates and HM leptin and adiponectin deserve some consideration. Specifically, the observation that maternal BMI positively associated with leptin is consistent with the existing literature [42,43]. The mechanism underlying this association remains under investigation. However, maternal BMI and HM leptin have both been correlated with maternal serum leptin in prior work [44,45]. In addition, mammary epithelial cells and adipose tissue have been reported to secrete leptin into milk [46,47,48]. Further work in this area will be important. The negative association between HM leptin and breastfeeding intensity is consistent with a prior study [10]. Maternal BMI, which negatively associates with breastfeeding intensity, may explain some of the observed association [49]. In general, the negative associations between HM leptin and adiponectin with breastfeeding intensity were modest in strength, aligning with inconsistent findings in prior studies [16,17,18,19]. The null associations identified for remaining covariates are largely consistent with prior literature [9,36,38,42,43,50].

Strengths of this study included a rigorous milk collection protocol including full breast expression standardized for time of day, consistent cold storage until analysis, standardized infant age, reproducible sample preparation, an analytical method validated for HM, adjustment for breastfeeding intensities and maternal and infant characteristics, and infant anthropometry obtained by trained staff, in duplicate, longitudinally, and at similar ages.

This study also had several limitations. The primary limitation is that our measures of infant eating behavior, including breastfeeding intensity, were by maternal report, which may introduce bias, and the only objective measurement of infant sucking occurred at only a single feeding. Second, the study may not have been sufficiently powered to detect small effect sizes. Third, the sample was based on a convenience method, which may introduce selection bias and limit the generalizability of the results. Fourth, some mother–infant dyads were excluded due to missing data, which could impact the representativeness of the sample. Fifth, maternal pre-pregnancy weight was self-reported, which could introduce recall bias. Sixth, milk hormones were assessed at only a single age point. However, the two-month age point was selected to assure mature milk and optimize the likelihood of breastfeeding exclusivity. Seventh, we did not collect 24 h milk volume, which would allow for the estimation of the daily intake of milk hormones. Eighth, our estimation of breastfeeding intensity was based on maternal reporting of the number of feedings per day, which did not account for the volume per feeding and therefore may have introduced measurement error. However, the large majority of our sample was exclusively breastfed, which limits the degree of error introduced. Ninth, the study required that infants be capable of feeding from an artificial nipple to enable measurement of sucking, which excluded from eligibility infants who exclusively fed from the breast. However, this group is the minority in the population [49]. Tenth, the instrumented nipple could have altered infant eating behavior. Eleventh, we did not collect detailed information on maternal dietary intake in the time period contemporaneous with HM sample collection. Future studies should consider more comprehensive phenotyping of maternal factors that could contribute to HM leptin and adiponectin.

## 5. Conclusions

In summary, HM leptin was predicted by maternal BMI, but no other maternal or infant characteristics tested were significantly associated with either leptin or adiponectin. HM leptin and adiponectin were each positively independently associated with markers of infant adiposity at later, but not earlier, ages. These results may partially explain associations between maternal and child adiposity. HM adiponectin may promote a pattern of feeding characterized by longer, less frequent feeds, but otherwise neither leptin nor adiponectin associated with any of the eating behaviors measured at any age. HM leptin and adiponectin may contribute to weight gain in early infancy, but the effect does not appear to be mediated substantially by infant eating behaviors. Future work requires the use of rigorous methods of HM hormone and infant body composition measurements and objective markers of infant appetite and breastfeeding intensity.

## Figures and Tables

**Table 1:** Cohort characteristics and correlations of milk hormones, eating behaviors, and anthropometry with potential covariates.

		HM Hormones		Anthropometry								Eating Behaviors						
		Leptin (ng/mL)	Adiponectin (ng/mL)	2m		4m		6m		∆ WAZ 2–6 Months	∆ WLZ 2–6 Months	2m						
				WAZ	WLZ	WAZ	WLZ	WAZ	WLZ			Feed Freq	Feed Dur	EF	FR	GA	Suck Freq (Hz)	Burst Dur (s)
Characteristics	M (SD)	0.41 (0.27)	14.31 (17.33)	−0.21 (0.86)	0.06 (0.98)	−0.22 (0.90)	0.07 (0.87)	−0.11 (1.00)	0.35 (0.98)	0.03 (0.13)	0.07 (0.19)	7.6 (0.8)	2.7 (1.1)	4.5 (0.4)	2.1 (0.6)	3.9 (0.9)	1.53 (0.27)	37.67 (34.53)
Maternal age (years) (Mean ± SD)	31.5 (5.0)	−0.09	−0.25 *	−0.08	−0.07	−0.14	−0.06	−0.19	−0.13	−0.20	−0.07	0.14	−0.13	−0.24 *	−0.15	−0.22	−0.04	0.13
Gestational Diabetes (Yes = 1, No = 0)	0.11	−0.11	−0.13	−0.13	−0.02	−0.14	−0.04	−0.14	−0.01	−0.03	−0.01	0.11	−0.11	−0.16	0.00	−0.08	−0.14	0.11
Pre-pregnancy BMI (kg/m^2^) (Mean ± SD)	27.2 (5.9)	0.38 *	0.11	−0.01	−0.04	0.09	0.12	0.18	0.25 *	0.35 *	0.37 *	−0.13	0.10	0.08	−0.05	0.19	0.12	0.16
Maternal education (high = 3, medium = 2, low = 1)	2.2 (0.8)	−0.18	−0.20	0.06	−0.29 *	0.03	−0.12	−0.02	−0.15	−0.11	0.18	−0.03	−0.22	−0.34 *	−0.37 *	−0.36 *	−0.14	−0.10
Infant age (months) ^a^ (Mean ± SD)	2.2 (0.4)	−0.12	−0.14	0.02	−0.01	–	–	–	–	–	–	0.03	−0.08	0.13	−0.15	−0.31	−0.11	0.18
Gestational age (weeks) (Mean ± SD)	39.4 (1.05)	−0.24	−0.16	0.12	−0.02	0.03	−0.15	−0.02	−0.17	−0.23	−0.18	−0.04	−0.10	0.22	0.15	0.04	0.09	−0.15
Birthweight (kg) (Mean ± SD)	3.43 (0.36)	−0.06	0.02	0.61 *	0.24	0.47 *	0.21	0.44 *	0.31 *	−0.12	0.11	−0.18	−0.01	0.19	−0.22	0.17	0.15	−0.08
Infant Sex (Female = 1, Male = 0)	0.56	0.02	−0.03	−0.07	−0.06	−0.07	−0.08	−0.13	−0.28 *	−0.13	−0.32 *	0.04	−0.20	−0.31 *	−0.09	−0.36 *	−0.09	−0.21
Breastfeeding Intensity (Mean ± SD)	0.91 (0.22)	−0.25 *	−0.25 *	−0.03	−0.02	−0.06	−0.0	−0.07	0.02	−0.08	0.05	0.03	−0.30 *	−0.06	−0.05	0.04	−0.10	0.06

* *p* < 0.05; ^a^ Age at human milk sample collection. Abbreviations: HM (Human Milk), WAZ (weight-for-age z-score), WLZ (weight-for-length z-score), Feed Freq (Feeding Frequency), Feed Dur (Feeding Duration), EF (Enjoyment of Food), FR (food responsiveness), GA (General Appetite), Suck Freq (Sucking Frequency), Burst Dur (Burst Duration).

**Table 2:** Associations of leptin and adiponectin with anthropometry in linear regression models.

	2m		4m		6m		∆ WAZ 2–6 Months	∆ WLZ 2–6 Months
	WAZ 2m β (SE)	WLZ 2m β (SE)	WAZ 4m β (SE)	WLZ 4m β (SE)	WAZ 6m β (SE)	WLZ 6m β (SE)	β (SE)	β (SE)
Adjusted only for breastfeeding intensity								
Leptin (ng/mL)	−0.0124 (0.404)	0.524 (0.456)	0.253 (0.422)	0.802 (0.399) *	0.771 (0.463)	1.166 (0.439) *	0.182 (0.059) *	0.149 (0.086)
Adiponectin (ng/mL)	0.007 (0.006)	0.007 (0.007)	0.009 (0.006)	0.007 (0.006)	0.014 (0.007)	0.016 (0.007) *	0.002 (0.001)	0.002 (0.001)
Fully adjusted ^a^								
Leptin (ng/mL)	0.172 (0.323)	0.368 (0.447)	0.401 (0.376)	0.802 (0.399)	0.913 (0.419) *	1.22 (0.467) *	0.133 (0.064) *	0.060 (0.088)
Adiponectin (ng/mL)	0.006 (0.005)	0.004 (0.007)	0.009 (0.006)	0.007 (0.006)	0.013 (0.007) *	0.014 (0.007) *	0.001 (0.001)	0.001 (0.001)

* *p* < 0.05; All models are adjusted for breastfeeding intensity; ^a^ Adjusted for the cohort characteristics previously identified in correlation analyses reported in Table 1 as significantly associated with each outcome. Abbreviations: WAZ (weight-for-age z-score), WLZ (weight-for-length z-score).

**Table 3:** Associations of leptin and adiponectin with infant eating behaviors in linear regression models.

	Feeding Frequency β (SE)	Feeding Duration β (SE)	Enjoyment of Food β (SE)	Food Responsiveness β (SE)	General Appetite β (SE)	Burst Duration β (SE)	Sucking Frequency β (SE)
Adjusted only for breastfeeding intensity							
Leptin (ng/mL)	0.153 (0.361)	0.420 (0.521)	0.064 (0.200)	−0.101 (0.290)	0.061 (0.437)	−8.768 (16.209)	0.055 (0.125)
Adiponectin (ng/mL)	−0.008 (0.006)	0.029 (0.013) *	−0.003 (0.003)	0.001 (0.004)	0.010 (0.007)	−0.159 (0.254)	0.002 (0.002)
Fully adjusted ^a^							
Leptin (ng/mL)	0.153 (0.361)	0.420 (0.521)	−0.007 (0.187)	−0.235 (0.274)	−0.118 (0.393)	−8.768 (16.209)	0.055 (0.125)
Adiponectin (ng/mL)	−0.008 (0.006)	0.029 (0.013) *	−0.005 (0.003)	−0.002 (0.004)	0.006 (0.006)	−0.159 (0.254)	0.002 (0.002)

* *p* < 0.05; All models are adjusted for breastfeeding intensity; ^a^ Adjusted for covariates significantly associated with each outcome.

## Data Availability

The data presented in this study are openly available in Deep Blue at https://deepblue.lib.umich.edu/data (accessed on 1 April 2026).

## Supplementary Materials

The following supporting information can be downloaded at: https://www.mdpi.com/article/10.3390/nu18081203/s1, Table S1: Differences Between Analytic Sample and Sample Excluded Due to Missing Data; Table S2: Associations of Leptin and Adiponectin with LAZ in Linear Regression Models; Table S3: Associations of Leptin and Adiponectin with Eating Behaviors at Ages 4 and 6 Months in Linear Regression Models.
