# Supplementary material for: The Association of Human Milk Appetite-Regulating Hormones with Infant Growth and Eating Behaviors to Age Six Months
Source: Nutrients. 2026 Apr 10;18(8):1203. doi: 10.3390/nu18081203 (PMC13118950; doi:10.3390/nu18081203)
Supplement: Supplementary file 1 [file nutrients-18-01203-s001.zip › nutrients-4195703-supplementary.pdf]

Supplementary Materials

Additional Information on Eating Behaviors

Mothers were asked to report how many times in a 24 hour period, in the last seven days, they fed their infant (any type of feeding) on a 7-point scale (1=1-2, 3=3, 4=4, 5=5, 6=6, 7=7, 8=8 or more), which we considered to be feeding frequency. Mothers also reported on a 6-point scale how long the average breastfeeding lasted (1=<10 minutes, 2=10-19, 3=20-29, 4=30-39, 5=40-49, 6=⩾50), which we considered to be feeding duration. At age 2 months, mothers responded to the question "In the past 7 days, how often was your baby fed each food listed below?" [25].

Table S1. Differences between analytic sample and sample excluded due to missing data

	Analytic sample N=70	Excluded N=214	p-value for test of difference
Maternal Age (years) (Mean ± SD)	31.5 ± 5.0	31.1 ± 5.0	0.61
Gestational Diabetes (GDM), n (%)	7 (10%)	14 (7%)	0.37
Pre-pregnancy BMI (kg/m²) (Mean ± SD)	27.2 ± 5.9	28.0 ± 7.6	0.46
Pre-pregnancy weight status, n (%)			0.43
Normal (BMI ≥18.5 <24.9)	31 (44%)	89 (41%)	
Overweight (BMI ≥25<29.9)	16 (23%)	45 (21%)	
Obese (BMI ≥30)	21 (30%)	62 (29%)	
Data Not Available	2 (3%)	19 (9%)	
Maternal Education, n (%)			0.07
Less than 4-year Undergraduate Degree	16 (23%)	79 (37%)	
Undergraduate Degree	26 (37%)	56 (26%)	
Graduate Degree	28 (40%)	79 (37%)	
Maternal Race/Ethnicity, n (%)			0.14
White, non-Hispanic	51 (73%)	147 (69%)	
Black, non-Hispanic	6 (9%)	32 (15%)	
Hispanic, any race	1 (1%)	11 (5%)	
Other	12 (17%)	22 (10%)	
Gestational Age (weeks) (Mean ± SD)	39.4 ± 1.0	39.5 ± 1.1	0.7
Birthweight (kg) (Mean ± SD)	3.43 ± 0.36	3.45 ± 0.42	0.77
Infant Sex, n (%)			0.24
Female	39 (56%)	102 (48%)	
Male	31 (44%)	112 (52%)	
Breastfeeding Intensity (Mean ± SD)	0.91 ± 0.22	0.65 ± 0.45	<0.0001

Table S2. Associations of leptin and adiponectin with LAZ in linear regression models

Infant age	2m	4m	6m	Δ LAZ
	LAZ	LAZ	LAZ	2–6 months
Means (SD)	−0.243 (0.904)	−0.336 (1.014)	−0.505 (1.021)	−0.062 (0.161)
	β (SE)	β (SE)	β (SE)	β (SE)
Adjusted only for breastfeeding intensity				
Leptin (ng/mL)	−0.458 (0.422)	−0.555 (0.473)	−0.203 (0.476)	0.061 (0.074)
Adiponectin (ng/mL)	0.002 (0.006)	0.006 (0.007)	0.003 (0.007)	0.000 (0.001)
Fully adjusted[a]				
Leptin (ng/mL)	−0.171 (0.348)	−0.404 (0.427)	−0.070 (0.448)	0.061 (0.072)
Adiponectin (ng/mL)	0.004 (0.005)	0.005 (0.007)	0.002 (0.007)	0.000 (0.001)

All models adjusted for breastfeeding intensity; [a]Adjusted for the cohort characteristics previously identified as significantly associated with each outcome.
Abbreviations: LAZ (length-for-age z-score)

Table S3. Associations of leptin and adiponectin with eating behaviors at ages 4 and 6 months in linear regression models

	Feeding Frequency		Feeding Duration		Enjoyment of Food		Food Responsiveness		General Appetite		Burst Duration		Sucking Frequency	
Infant age	4m	6m	4m	6m	4m	6m	4m	6m	4m	6m	4m	6m	4m	6m
Mean (SD)	7.373 (1.042)	7.552 (0.764)	2.347 (1.032)	2.265 (1.016)	4.493 (0.431)	4.466 (0.439)	2.112 (0.576)	2.013 (0.646)	3.846 (0.833)	3.806 (0.821)	47.272 (61.643)	52.249 (72.418)	1.617 (0.255)	1.596 (0.252)
	β (SE)	β (SE)	β (SE)	β (SE)	β (SE)	β (SE)	β (SE)	β (SE)	β (SE)	β (SE)	β (SE)	β (SE)	β (SE)	β (SE)
Adjusted only for breastfeeding intensity														
Leptin (ng/mL)	−0.474 (0.489)	−0.262 (0.363)	1.305 (0.756)	1.439 (0.797)	0.262 (0.200)	−0.054 (0.210)	−0.027 (0.273)	−0.176 (0.307)	0.567 (0.407)	0.396 (0.383)	−21.84 (29.36)	−13.47 (34.59)	0.227 (0.118)	0.181 (0.116)
Adiponectin (ng/mL)	−0.017 (0.007)*	-0.001 (0.006)	0.026 (0.031)	0.015 (0.029)	−0.002 (0.003)	0.002 (0.003)	0.000 (0.004)	0.002 (0.005)	0.016 (0.010)	0.013 (0.006)*	−0.133 (0.457)	0.806 (0.527)	0.003 (0.002)	0.003 (0.002)
Fully adjusted[a]														
Leptin (ng/mL)	−0.653 (0.480)	−0.246 (0.373)	1.305 (0.756)	1.088 (0.771)	0.262 (0.200)	−0.087 (0.200)	−0.143 (0.263)	−0.138 (0.308)	0.369 (0.371)	0.258 (0.329)	−14.36 (28.13)	−13.47 (34.59)	0.048 (0.129)	0.181 (0.116)
Adiponectin (ng/mL)	−0.021 (0.007)*	−0.001 (0.006)	0.026 (0.031)	0.006 (0.028)	−0.002 (0.003)	0.001 (0.003)	−0.002 (0.004)	0.001 (0.005)	0.006 (0.010)	0.009 (0.005)	0.054 (0.439)	0.806 (0.527)	0.002 (0.002)	0.003 (0.002)

*$p<0.05$; All models adjusted for breastfeeding intensity; [a]Adjusted for covariates significantly associated with each outcome.
